# Towards comprehensive understanding of bacterial genetic diversity: large-scale amplifications in *Bordetella pertussis* and *Mycobacterium tuberculosis*

**DOI:** 10.1099/mgen.0.000761

**Published:** 2022-02-10

**Authors:** Jonathan S. Abrahams, Michael R. Weigand, Natalie Ring, Iain MacArthur, Joss Etty, Scott Peng, Margaret M. Williams, Barret Bready, Anthony P. Catalano, Jennifer R. Davis, Michael D. Kaiser, John S. Oliver, Jay M. Sage, Stefan Bagby, M. Lucia Tondella, Andrew R. Gorringe, Andrew Preston

**Affiliations:** ^1^​ Department of Biology and Biochemistry and Milner Centre for Evolution, University of Bath, Bath, UK; ^2^​ Division of Bacterial Diseases, Centers for Disease Control and Prevention, Atlanta, Georgia, USA; ^3^​ Nabsys 2.0, Providence, RI 02809, USA; ^4^​ Public Health England, Porton Down, Salisbury, UK

**Keywords:** *B. pertussis*, genome structure, duplications, genetic diversity, amplifications

## Abstract

Bacterial genetic diversity is often described solely using base-pair changes despite a wide variety of other mutation types likely being major contributors. Tandem duplication/amplifications are thought to be widespread among bacteria but due to their often-intractable size and instability, comprehensive studies of these mutations are rare. We define a methodology to investigate amplifications in bacterial genomes based on read depth of genome sequence data as a proxy for copy number. We demonstrate the approach with *Bordetella pertussis *, whose insertion sequence element-rich genome provides extensive scope for amplifications to occur. Analysis of data for 2430 *B. pertussis* isolates identified 272 putative amplifications, of which 94 % were located at 11 hotspot loci. We demonstrate limited phylogenetic connection for the occurrence of amplifications, suggesting unstable and sporadic characteristics. Genome instability was further described *in vitro* using long-read sequencing via the Nanopore platform, which revealed that clonally derived laboratory cultures produced heterogenous populations rapidly. We extended this research to analyse a population of 1000 isolates of another important pathogen, *Mycobacterium tuberculosis *. We found 590 amplifications in *M. tuberculosis *, and like *B. pertussis *, these occurred primarily at hotspots. Genes amplified in *B. pertussis* include those involved in motility and respiration, whilst in *M. tuberuclosis*, functions included intracellular growth and regulation of virulence. Using publicly available short-read data we predicted previously unrecognized, large amplifications in *B. pertussis* and *M. tuberculosis *. This reveals the unrecognized and dynamic genetic diversity of *B. pertussis* and *M. tuberculosis *, highlighting the need for a more holistic understanding of bacterial genetics.

## Data Summary

All data generated during this study are included in this published article and its supplementary information files. Illumina data for the 28 resolved genome isolates is available on the SRA (https://www.ncbi.nlm.nih.gov/sra) with accession numbers SRR9123572, SRR5829828, SRR9006092-3, SRR9123574, SRR9151823, SRR9006149, SRR5829737, SRR5829824, SRR5829749, SRR5829769/SRR5829803, SRR9006067, SRR9118395, SRR5829789/SRR5829798, SRR9118319, SRR9118314, SRR9118293, SRR9118269, SRR9118452, SRR5070923/SRR5514663, SRR5071090/SRR5514664, SRR9131605, SRR9131607, SRR9131663-5.SRR9151824.

Nanopore sequencing runs are deposited on the SRA under the project PRJNA604974. Code needed to reproduce the analysis in this study is contained on Github and is linked to throughout the text.

**Impact Statement**

Most studies of microbial genetic diversity have been limited to variations detected within short-read DNA sequencing. However, this excludes structural variation, which although widespread among bacteria has been largely overlooked with regards to contributions to microbial diversity. Here, we identify copy-number variations of large genomic regions as a previously unrecognized contribution to *B. pertussis* genomic diversity. We demonstrate a surprising plasticity to these structural variations, suggesting a dynamic mode of genome variation. This adds to the developing picture of *B. pertussis* variation. We expand our observations made with *B. pertussis* to *M. tuberculosis,* again identifying previously unrecognized CNVs. Most studies of bacterial genetic diversity have focused on small nucleotide changes (SNPs, indels) or wholesale acquisition of genes via horizontal gene transfer. Our study reveals structural variation as potentially widespread but poorly appreciated among bacteria. The effects of CNVs on bacterial phenotype is unclear, but we demonstrate that copy number changes can affect gene-expression patterns. A holistic view of bacterial genetic diversity should include all forms of variation, including structural variation and the growing accessibility to long-read DNA sequencing opens up this aspect to much wider study.

## Introduction

At the time of writing there are over 1.2 million bacterial whole-genome sequencing runs in the European Nucleotide Archive (ENA), the majority of which have not been analysed for large deletions, inversions or tandem duplications/amplifications (referred to simply here as amplifications), collectively known as structural variants. Despite this, there is a rich and diverse literature describing structural variants and their phenotypes including antimicrobial resistance [1, 2] and increased virulence [3, 4] – topics of major public health concern.

As many structural variants are formed through homologous recombination, bacterial species with highly repetitive genomes are likely to experience an increased burden of structural variants. In this study we focus primarily on *Bordetella pertussis *, the main causative agent of whooping cough, genomes of which have approximately 200 copies of IS*481* [5, 6]. Speciation of *B. pertussis* from a *B. bronchiseptica *-like ancestor was synonymous with the accumulation of insertion sequence (IS) elements and subsequent large-scale rearrangements and deletions, likely though homologous recombination between repeats. As a result, genomes of *B. pertussis* encode at least 1000 fewer genes than *B. bronchiseptica* [5].

Homologous recombination between repetitive IS elements still plays a major role in the genetics of *B. pertussis* and as such the species is described as having a plastic genome [5, 7, 8]. *B. pertussis* genomes experience deletions, inversions and amplifications of large tracts of DNA, although these distinct forms of structural variation have been described to widely varying levels. Deletions mediated by homologous recombination continue to streamline the genome in extant *B. pertussis* lineages and have enjoyed systematic description [8, 9]. Inversions have been similarly subject to description [10], and with IS having been found to influence the expression of neighbouring genes [11] it is likely that inversions are a novel source of variability in the *B. pertussis* transcriptome [11, 12]. The scale of analysis of genomic inversions has been catalysed by advances in long-read sequencing yet amplifications, in contrast, have been found only 11 times previously, primarily using techniques that predate whole-genome sequencing [13–19]. There exists now a wealth of whole-genome sequencing data suitable for studying amplifications, yet there has been no systematic investigation of their contribution to genomic diversity within the *B. pertussis* population. *B. pertussis* is therefore an attractive candidate for the prevalence of amplifications to be systematically analysed.

Homologous recombination is a ubiquitous process among bacteria and, as this process can lead to the formation of structural variants, these must be ubiquitous too. We aimed to describe the prevalence of these mutations in an unrelated species, *M. tuberculosis *. Compared to *B. pertussis *, *M. tuberculosis* has fewer repeats, and major differences in pathogenesis, global distribution and disease mortality/burden [20, 21]. Furthermore, in contrast to *B. pertussis *, the *M. tuberculosis* genome contains only approximately 30 copies of IS elements although it does also contain approximately 150 genes, which share high homology and contain Pro-Glu (PE genes) or Pro-Pro-Glu (PPE genes) motifs at their termini [22, 23]. Seven amplifications have been described previously for *M. tuberculosis* but, as with *B. pertussis *, were not found using a systematic analysis that searched for genome-wide structural variants [24–26].

Here we address this knowledge gap by leveraging the wealth of public short-read sequencing data to screen *B. pertussis* and *M. tuberculosis* isolates for evidence of amplification according to read depth. We detected hundreds of putative amplifications occurring primarily at ‘hotspot’ loci and varying in size up 600 kb. We further investigated the instability of these amplifications in *B. pertussis* over both short and long timescales using ultra-long Nanopore sequencing and phylogenetics, respectively. Such transient gene amplifications are an often-overlooked source of genetic variation in bacteria, and our study provides a blueprint to more widely describe both their structure and putative impact, which can include large numbers of protein-coding genes.

## Methods

### Sequence read mapping

Short-read data originating from the Illumina platform were retrieved from the National Centre for Biotechnology Information’s (NCBI) Sequence Read Archive (SRA). One run was chosen at random for each BioSample, totalling 2803 runs. Reads were mapped to the *B. pertussis* B1917 genome, which is broadly representative of the modern circulating isolates [27] (RefSeq ID: NZ_CP009751.1), using BWA [28] implemented in Snippy (version 4.3) (available: https://github.com/tseemann/snippy).

### Amplification prediction

CNVnator [29] was used to predict amplifications from read depth data generated from the mapping process. Statistical tests for significance within CNVnator discriminate high and low confidence calls. CNVnator predicts amplifications as genomic ranges (e.g. base 1000–2000), which is then processed into a range of genes (e.g. gene 10–20). To increase specificity of the pipeline, we implemented a very low *P*-value cut-off (*P*<0.0001). Abyzov *et al*. empirically tested CNVnator to determine that ratios of the average read depth to the standard deviation of 4–5 produce the best balance between sensitivity and specificity [29]. In accordance, samples exhibiting ratios <3 were discarded as amplification calls were unreliable on such variable data, as documented by Abyzov *et al*. [29]. Window length was optimized for each genome, testing window sizes 500–1000 bp at intervals of 100 bp to evaluate which gave a ratio closest to 4.5 as to minimize the effect of stochastic and/or artefactual fluctuations in read depth across the genome. Copy number estimates were rounded to the nearest 0.1. Code is available: https://github.com/Jonathan-Abrahams/Duplications

### Control data

As a negative control, short-reads were simulated from the B1917 reference genome using ART to simulate the error profile of Illumina HiSeq paired-end 150 bp data (-ss HS25 -p -l 150 f 20 m 200 s 10) [30]. Simulated reads were mapped back to the reference genome using Snippy and CNVnator was used to call any spurious amplifications, as described above [29]. As a positive control dataset, closed genome sequences from 25 isolates with manually resolved amplifications were used. This data was generated using a combination of PacBio and Illumina sequencing and optical mapping on the Argus or Nabsys HD platforms, as done previously [7, 31, 32].

### Association of amplifications with repeat regions

The association of predicted amplifications with repetitive sequences was tested. This was only possible in closed genome sequences and therefore only amplifications in these genomes were analysed. Closed genome sequences for isolates containing putative amplifications (excluding previously verified amplifications) were downloaded and the association of amplification boundaries with repeat sequences was determined using R and blast.

### Heatmap

The read depth-based predictions were hierarchically clustered based on the similarities of amplification profiles (including deletions) of samples using the R package Hclust. This therefore meant that isolates with similar complements of amplifications and deletions were clustered together on the heatmap, which was plotted using the R package Plotly (Plotly Technologies 2015).

### Networks

Overlapping gene content among amplifications was evaluated by constructing undirected network graphs, which quantified the relationships (edges) between each amplification (nodes). An edge was constructed between nodes if both amplifications had a 50 % overlap (non-reciprocal). Network analysis was undertaken in R using the Igraph package [33] and networks layout was generated by the Fruchterman algorithm [34].

### qPCR

Bacteria were grown on charcoal agar for 3 days at 37 °C before inoculation into Stainer–Scholte (SS) broth (Stainer and Scholte 1970) and grown overnight at 37 °C with shaking at 180 r.p.m.; these cultures were used to inoculate fresh media at an OD_600_=0.2. Bacterial cells were harvested (1 ml for DNA and 10 ml for RNA extraction) at OD_600_=1.1±0.1 by centrifugation (4000 ***g*** for 10 min) and resuspended in 700 µl of Tri-reagent (Invitrogen, ThermoFisher, Loughborough, UK), vortexed vigorously, and frozen at −80 °C. DNA was purified using QIAamp kit (Qiagen, Manchester, UK) in accordance with the manufacturer’s instructions. The concentration of DNA was determined using Qubit broad range DNA quantification kit (Fisher Scientific).

qPCR was run on a StepOne Real-time PCR System (Applied Biosystems, ThermoFisher) using TaqMan Universal PCR Master Mix (Applied Biosystems) in a total reaction volume of 20 µl with 100 pg of DNA and with primer and probe concentrations as described in Table 1. Biological triplicates and technical triplicates were run for each sample, apart from the initial screen of UK54 clones where only one biological sample was used for each clone. Reaction conditions were as follows: 10 min at 95 °C followed by 40 cycles of 15 s at 95 °C and 1 min at 60 °C. Copy number was quantified by using the 2^-ΔΔ*CT*^ method. Three biological repeats were used for determination of copy number in UK54.

**Table 1.** qPCR primers and probes used in addition to the determined optimal concentration

Name	expt	Sequence (5′ to 3′)	concn (nM)
CNV_fw	Copy number determination	TCTGGGGAGTCGAAAGCAAT	300
CNV_rv	Copy number determination	TCTTGAGGGTGGCGAAGAAT	900
CNV_probe	Copy number determination	FAM-ACGCCCCTTGCTGACGTCGC-BHQ	200
BP283_fw	Copy number determination	CAGGCACAGCACTATTGCG	500
BP283_RV	Copy number determination	GACGATTACCAGCGAGATTACGA	300
BP283_probe	Copy number determination	FAM-CCGCCATCGCAACCGTCGCATTCA-BHQ	200
CNV_fw	Gene expression	TCTGGGGAGTCGAAAGCAAT	300
CNV_rv	Gene expression	TCTTGAGGGTGGCGAAGAAT	300
RecA_fw	Gene expression	CGCGTCAAGGTGGTCAAGA	300
RecA_rv	Gene expression	CTGCCATACATGATGTCGAACTC	300
RecA probe	Gene expression	FAM-TGGCGCCGCCGTTCAAGC-BHQ	250
1736F	Gene expression	AAGACAAGCCCAAGCAATCG	300
1736R	Gene expression	TCACCACGCCATTGTTCGT	300
1736 probe	Gene expression	FAM-CGAGTACGCCTCCGATGCCACG-BHQ	250
1740F	Gene expression	TGCGCAATCACTCCTCCAT	300
1740R	Gene expression	AAGTCACGACATCGAGAAATTCAA	300
1744F	Gene expression	ATGCCGGATTCGACGACTT	300
1744R	Gene expression	CGGTCCTGGCGGATTTTC	300

To isolate RNA, nucleic acids were precipitated with ethanol, residual DNA was removed by incubation with 4U of Turbo DNase (Ambion, ThermoFisher) for 1 h at 37 °C, and RNA was purified using the RNeasy kit (Qiagen, Manchester, UK) in accordance with the manufacturer’s instructions. The concentration of RNA was determined using Qubit broad range RNA quantification kit (Fisher Scientific). RNA integrity was determined by agarose gel electrophoresis. Finally, RNA was confirmed as being DNA free by PCR using 50 ng of RNA as template in PCR with *recA*F and *recA*R primers. First-strand cDNA was synthesized using ProtoScript II (NEB) with 1 µg of total RNA as template and 6 µM random primers and incubated for 5 min at 25 °C, 1 h at 42 °C. The reaction was stopped by incubating at 65 °C for 20 min. cDNA was diluted 1/30 in H_2_O for use in qPCR.

RT-qPCR was run on an a StepOne Real-time PCR System using TaqMan Universal PCR Master Mix (Applied Biosystems) or SyberGreen Turbo Master mix (Applied Biosystems), in a total reaction volume of 20 µl with primers at 300 nM and probes at 250 nM. Triplicate reactions were run for each sample. Reactions conditions were as follows: 95 °C for 10 min and 40 cycles of 95 °C for 15 s and 1 min at 60 °C. The housekeeping gene *recA* was used as a stably expressed control gene. The ∆CT and ∆∆CT were calculated by determining the difference between the reference condition and experimental condition. Relative expression was represented as fold change (fold change=2^-∆∆CT^). Significance was determined with one-way ANOVA.

### Optical mapping

Genomic DNA isolation from *Bordetella pertussis* isolates D236, D800, H624, J085, J196 and J321 was performed at the CDC according to a Nabsys solution-based protocol modified from the bacterial DNA protocol for AXG 20 columns and Nucleobond Buffer Set III (Macherey-Nagel, Bethlehem, PA). Purified DNA was sent to Nabsys for nicking, tagging, coating and data collection on an HD-Mapping instrument. Nicking enzyme Nb.BssSI (NEB) was used for isolate D236 and the nicking enzyme combination Nt.BspQI/Nb.BbvCI (NEB) was used for isolates D800, H624, J085, J196 and J321. Resulting *de novo* assembled HD maps, raw data and data remapped to PacBio *de novo* assemblies were provided by Nabsys for further analysis and sequence assembly comparisons at the CDC using NPS analysis (v1.2.2424) and CompareAssemblyToReference (v1.10.0.1).

### Nanopore sequencing

*B. pertussis* isolate UK54 bacteria were stored at −80 °C in PBS/20 % glycerol at the University of Bath. Bacteria were grown for 72 h at 37 °C on charcoal agar (Oxoid) plates. Harvested cells were resuspended in 10 ml SS broth to an OD_600_ of 0.1 and grown overnight. At approximately OD_600_ 1.0, cultures were diluted in 50 ml SS broth to an OD_600_ of 0.1 and grown to OD_600_ 1.0. Bacteria were centrifuged at 13 000 ***g*** for 5 min and processed for gDNA extraction using the protocol available from dx.doi.org/10.17504/protocols.io.mrxc57n. The rapid adaptor (SQK-RAD004) Nanopore library preparation steps were included, adapted for sequencing of very long gDNA molecules.

DNA was sequenced for 48 h on GridION or MinION sequencers using R9.4 flow cells. Base-calling was performed with Guppy (V2.1.3 or V3.2.1) using the ‘fast’ Flip-flop model. Reads spanning the amplification locus were identified using Blastn alignment with a minimum query length coverage of 90 % for the 16 kb amplification locus and 10 % for the single-copy flanking regions (~1 kb).

### Phylogenetics

To investigate the phylogenetic relationship between isolates containing amplifications, a core-genome SNP alignment was created using Snippy (available: https://github.com/tseemann/snippy). An initial phylogenetic tree was constructed using IQtree2 [35]. Ultra-fast bootstrapping (UFboot2) was used as implemented in IQtree2 and as per recommendations, branches with UFBoot2 scores <95, approximately equivalent to 80 % standard bootstrap support, were collapsed [36, 37]. The model that fit the data the best was chosen using ModelFinder, as implemented in IQtree2 [38]. The optimal model for both trees was ‘TVM+F+ASC’. Itol [39] was used to display the tree. A subclade of the tree was plotted to aid visualization.

## Results

The US Centers for Disease Control and Prevention (CDC) conducts routine and enhanced surveillance of pertussis, which includes whole-genome sequencing of *B. pertussis* clinical isolates using the PacBio and Illumina platforms. Including both retrospectively and prospectively sequenced samples, 725 isolates were sequenced during the years 2014–2018 and their genome sequences assembled. Forty four assemblies could not be resolved. The sequence data contained evidence of amplifications, primarily, increased read depth coverage localized to discrete genomic regions of approximately 2–3 × the expected depth. Sequence data alone was incapable of resolving assembly of these regions but optical mapping of high-molecular weight DNA confirmed that the high read depth resulted from tandem amplifications. Amplifications were identified in 28 isolates, which each contained one amplification, including those used in the production of pertussis vaccines [17]. Conflicting genome orders from multiple data sources and/or inadequate coverage of the amplification junction when reads were mapped to the hypothesized genome order led to the genome sequences of the remaining 16 isolates not being closed using the manual assembly method. The identified amplifications ranged from 15 to 197 kb in length and involved hundreds of genes. Similar regions were amplified in multiple isolates although not always with conserved breakpoints. The accurate assembly of these genomes required manual resolution, using data from short-read, long-read and optical mapping sources. Using the manually resolved dataset as a benchmark, we sought to develop a prediction and screening algorithm to identify amplifications within the public repository of *B. pertussis* genome sequence data on the Sequence Read Archive (SRA) using a scalable and automated approach.

### Read depth as a proxy for copy number

**Fig. 1.**
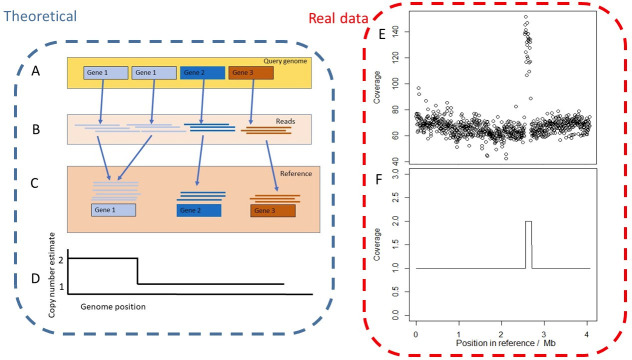
Schematic overview of prediction of amplifications from sequencing read depth. In the theoretical example (purple box, left), the query strain contains a tandem amplification of gene 1 whilst gene 2 and 3 are at single copy (a). Short-reads from the query strain are generated (b) and mapped to the reference genome, that contains all genes at single copy (c). Reads from both copies of gene 1 in the query strain map to this locus in the reference sequence and thus twice as many reads map to this gene compared to genes 2 and 3. This data must be processed to avoid technical bias, the pipeline processes read coverage data into estimates of copy number (d). Using an example with real data (red box, right) the strain SAMN08200079 was analysed. Read coverage was plotted to reveal an amplification at ~1.4 Mb (e, analogous to theoretical graph c), which was statistically analysed using our pipeline (f, analogous to theoretical graph d).

We mapped short-read data from each query strain to a reference genome sequence and used read depth as a proxy for copy number of individual genomic regions, namely protein-coding genes (Fig. 1). If the query strain contained two copies of a locus that was present at single copy in the reference genome, twice as many mapped reads would be detected at that locus. Conversely, a gene deletion in the query strain produces zero read depth at that locus in the reference. Due to a combination of biases and stochasticity read depth coverage fluctuates during whole-genome sequencing [40, 41] and therefore must be normalized and loci with greater read depth coverage analysed for statistical significance. We carried out this process using the tool CNVnator [29].

The performance of our amplification prediction method was tested on two datasets: a simulated negative control and the manually resolved amplifications contained in the CDC strains described above. For the negative control, reads were simulated for the reference strain B1917 (containing no amplifications) and mapped to its sequence. As expected, all genes were predicted at single copy number, with no false positives or negatives.

For the manually resolved amplifications, a prediction was considered correct if it had an 80 % reciprocal overlap with the true amplification. Out of the 28 isolates, three were excluded from further analysis. One isolate failed quality-control tests due to read-depth noise that was too erratic to be normalized. Two isolates were excluded due to divergent gene orders compared to the reference at the amplification loci, which meant the accuracy of predictions in these samples could not be judged fairly.

Our method was used to predict amplifications in the remaining 25 high-quality isolates with the same gene order as the reference (Table S1, available in the online version of this article). The amplifications for 23 of the 25 resolved genomes (Fig. S1) were predicted correctly and with excellent breakpoint accuracy. In two of these isolates the amplification was predicted correctly but as a major fragment (which satisfied the 80 % reciprocal overlap rule needed to be a true positive) and a minor fragment, which was counted as a false positive. One isolate had a second amplification predicted at a different locus and on manual inspection, whilst there was a rise in coverage at this locus, consultation of the optical maps did not evidence an amplification here and as such this was counted as a false positive. Thus, the pipeline correctly predicted, and with good breakpoint accuracy, the amplifications for 20 of the 27 isolates with high-quality data (74 %), with two further amplifications correctly predicted (11 %) but each having a major and minor fragment and with two isolates excluded for having divergent gene orders at the amplification locus. Our results were comparable to other implementations of CNVnator and read-depth prediction tools [29, 42].

### Amplifications as a source of genetic diversity

**Fig. 2.**
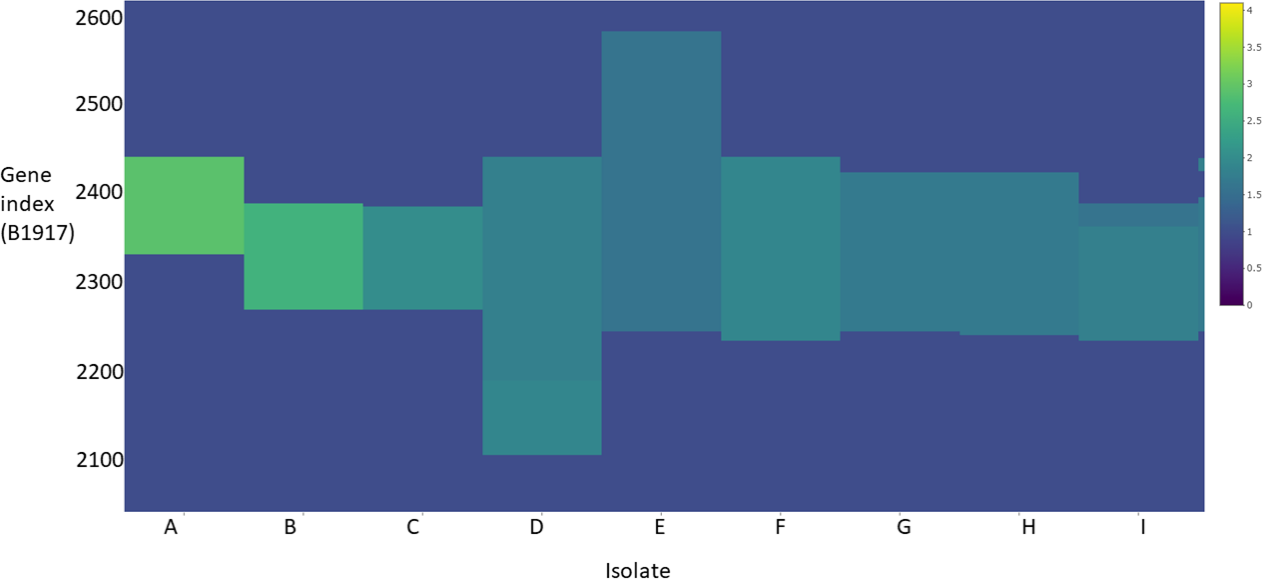
Heatmap containing the copy-number states gene-by-gene index (*Y* axis) of 9 isolates (A to I) belonging to network 1 (*X* axis). Colour scale (*Z* axis) indicates the copy number of each gene. A legend of the colour scale is on the far right.

Short-read sequence data from the SRA for 2709 *B. pertussis* isolates were mapped to the reference genome, B1917. Of these, 94 exhibited <30 × average coverage and 185 had highly erratic read coverage. The final test dataset therefore comprised 2430 *B. pertussis* isolates. Of these, 1711 had all genes predicted at single copy, 528 isolates had at least one putative deletion and 191 isolates had at least one amplification (8 % of the studied isolates), relative to B1917. 272 amplifications were observed in these 191 isolates, meaning some isolates contained multiple amplifications. Computed copy-number estimates were visualized with a heatmap where it became apparent that particular loci were amplified in multiple isolates (Fig. 2). Consistent with our observations in the resolved dataset and previous reports [17, 19, 43], amplifications at these loci varied in length between isolates, with differing breakpoints. It was found that genes at the start/end of the predicted amplifications were significantly associated (*P*<0.05) with repeat sequences (such as IS481 and rRNA) when compared to all genes in the B1917 genome (Fig. S2). This supported the validity of the predictions and suggested homologous recombination was the likely mechanism of the creation of these mutations.

### Most amplifications occur at hotspots

It was clear that the majority of amplifications were overlapping at a small number of loci (Fig. 2). There appeared to be genes that were present in the vast majority of amplifications at these loci and genes that appeared less commonly amplified on the periphery. These qualities matched the limited description of bacterial amplification hotspots in the literature [2, 25], which found that positive selection drove the formation of hotspots. In particular, Hjort *et al*. found that all amplifications (with an approximate average size of 100 kb) in a hotspot were maintained by positive selection for increased copy number of a single gene involved in antibiotic resistance [44]. We hypothesized that the hotspots described here were also maintained by positive selection. Thus we sought to describe the core genes in each hotspot, the genes most likely to be under positive selection [2].

The pairwise overlap between all amplifications at each region was recorded and network graphs were constructed between amplifications (‘nodes’) that were connected by at least 50 % content overlap (‘edges’). The 272 identified amplifications formed 24 network graphs, representing 24 distinct genomic loci (Table S2).

Eleven loci had amplifications in three or more isolates and contained 254/272 (93 %) of the predicted amplifications (Table 2, Fig. S3), we formally defined these loci as hotspots. To investigate genes within each hotspot that may have been under positive selection, we defined the hotspot ‘core’ as those genes present in at least 90 % of amplifications of that locus. To ensure accuracy, the core was calculated only for hotspots observed in at least ten strains.

**Table 2.** Eleven hotspot loci contained 254/272 (93 %) of the predicted amplifications and are described here. Columns are, from left to right: the hotspot name (ordered by frequency), the number of strains in which each hotspot was observed, median number of genes in each amplification, median start gene, median end gene and mean copy number. Median start/end columns refer to the locus tags in the reference genome, B1917. Expanded on in Table S3

Hotspot name	Frequency	Median length (genes)	Median start	Median end	Mean copy no.
**1**	102	106	B1917_RS12140	B1917_RS12755	1.6
**2**	57	82	B1917_RS15100	B1917_RS15490	1.7
**3**	21	80	B1917_RS07175	B1917_RS07660	1.68
**4**	18	20	B1917_RS00010	B1917_RS00130	1.35
**5**	13	67	B1917_RS19230	B1917_RS19625	1.93
**6**	11	75	B1917_RS05505	B1917_RS05935	1.6
**7**	8	49	B1917_RS04185	B1917_RS04430	1.88
**8**	8	74	B1917_RS09665	B1917_RS10290	1.82
**9**	7	13	B1917_RS19965	B1917_RS10580	2.49
**10**	6	23	B1917_RS19465	B1917_RS19565	1.32
**11**	3	45	B1917_RS01035	B1917_RS01300	1.63

Some isolates had multiple, separate amplifications at the same hotspot region. This may have been due to a complex mixture of structural variations affecting the same locus, such as nested amplifications [32] or the locus being disrupted by inversions. It is also possible that, as detected in two cases of the benchmarking experiment, a single gene was predicted to be single copy, splitting an amplification into two separate regions of higher copy number.

For the most frequently observed hotspot, hotspot 1, 71 genes were in the core and included genes encoding the flagella machinery (*flg* and *fli* operons in addition to *flaG*). This hotspot also contained genes predicted to encode an ABC-type amino acid transporter (B1917_RS12575, B1917_RS12580 and B1917_RS12585), the periplasmic substrate binding domain of which is homologous to annotated transporters of cystine in the Conserved Domain Database. Amplification at hotspot 2 was observed in 57 isolates, with 31 core genes included in at least 90 % of the amplifications at this region. These included the *nuo* operon, which encodes components of NADH:ubiquinone oxidoreductase [45], the first large complex of the electron transport chain, amplification of which perhaps enhanced metabolism.

### Plasticity of amplifications on a global scale and during *in vitro* growth

Previous research found that distinct lineages of cells under positive selection for increased copy number of the same locus generated the same hotspot effect described here [1, 24]. This may explain the observed diversity in the gene contents of amplifications at hotspots. Alternatively, this may have arisen from a single ancestral mutation that had been remodelled by further structural variants in related isolates. To distinguish between these scenarios, we investigated the phylogenetic relationships between isolates having amplifications at the same hotspots and observed that they had distant phylogenetic relationships. This suggested that amplifications arose independently in multiple different isolates (Fig. S4).

Underlying the phylogenetics of amplifications are the genome dynamics of *B. pertussis* over short time scales. We observed that amplifications were often predicted with non-integer copy numbers (1.2 or 2.2 etc), including among the benchmark dataset of manually resolved amplifications (Fig. S5). Non-integer copy-number predictions might arise from sequence data deriving from laboratory cultures in which the locus copy number varies among cells, with the predicted value being the average read depth among that population. To investigate this, we exploited the tractable size of one relatively small amplification. The genome of UK54 (SAMEA1920853) was predicted to have a 16 kb amplification at a copy number of 4.1; short enough to observe the entire amplification locus in a single sequence read on the Nanopore platform, assuming that each copy occurred in tandem as observed in both our data and previous reports [17, 43]. The amplification was part of hotspot 9, which comprised six other amplifications, one of which was also predicted at a copy number >2 (3.3, Isolate SAMN11822098).

**Fig. 3.**
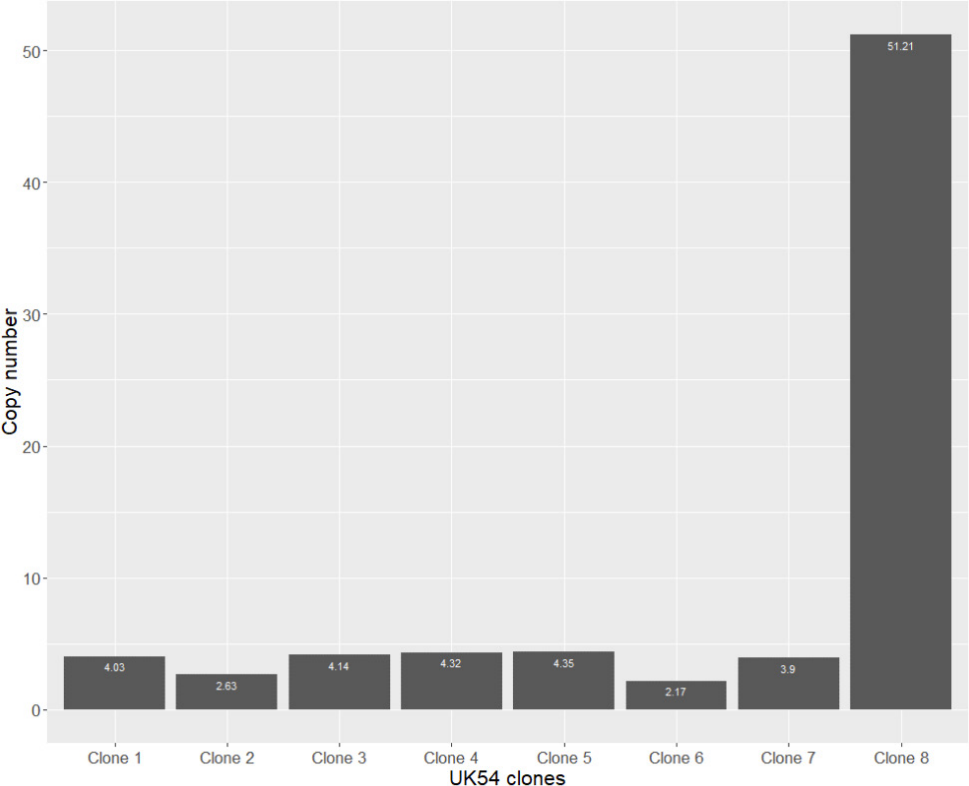
Quantification of amplification copy number of 8 clones of UK54 (*X* axis) by qPCR demonstrated a range of copy numbers from 2.17 to 51.21 (*Y* axis). UK54 was predicted to have an 16 kb amplification at the copy number of 4.1. Eight clones of UK54 were screened for their copy number of this locus (*X* axis) and their copy number was determined (*Y* axis). For clarity, the exact copy number that was determined is noted in white text in each bar. A range of copy numbers could be seen, from 2.17 to 51.21.

We picked eight single colonies of UK54 and passaged them once by growth on agar and then once during broth growth. Each of these clonal populations were theoretically derived from a single bacterium. The copy number at the amplification locus in each of the resulting clones was estimated using qPCR (Fig. 3) and ranged from 2.2 (clone 6) to 51.2 (clone 8).

**Fig. 4.**
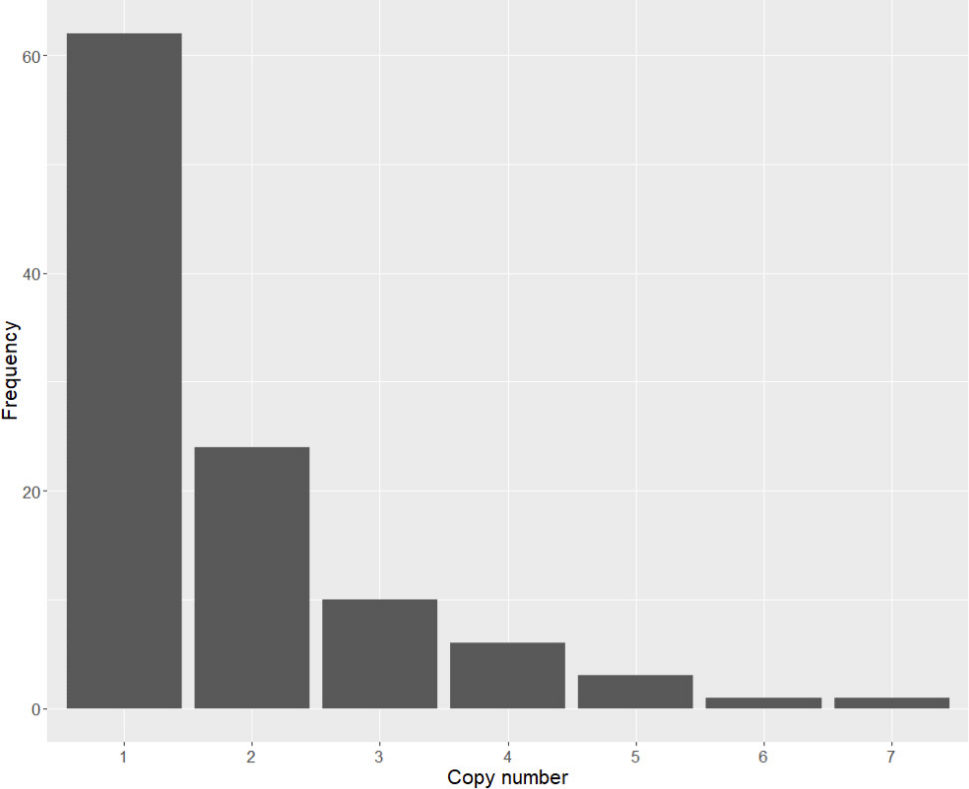
Nanopore sequencing of UK54 clone 8 revealed reads with between one and seven copies of the amplified locus (*X* axis) in varying frequencies (*Y* axis) within a single culture. No reads were observed that spanned the full locus, so the true copy number for these reads is unknown.

We sequenced UK54 clone 4 and 8, with copy numbers of 4.3 and 51.2, respectively, using the ultra-long-read Nanopore protocol (Fig. 4). For clone 4, we observed individual reads containing tandem arrays of either two or five copies of the locus with flanking DNA on both sides (i.e. the complete amplification was captured on a single read). For clone 8, which exhibited a copy-number estimate of 51 by qPCR (corresponding to a predicted amplification length of 816 kb), no reads spanning the entire amplification locus (i.e. the amplification locus with flanking DNA on each side) were identified, presumably due to its extreme length. However, reads containing up to seven copies of the locus, without flanking regions, were identified. Relaxing the blastn alignment parameters from a 90 % minimum query length of the amplification locus to 50 % identified a maximum of nine copies of the locus present on a single read with incompletely sequenced copies at each end. Consistent with the copy-number prediction from qPCR, the read depth at this locus for UK54 clone eight from the Nanopore data was approximately 60 × higher than the genome average, strongly supporting the very high copy-number estimate for this locus in this clone.

Thus, cells containing different copy numbers arose during *in vitro* growth from a single bacterium to the culture from which the gDNA was extracted, which we estimate to equate to approximately 25 generations. This strongly suggested that amplification copy number was plastic, and variation in copy number occurred over short time scales.

### Copy number correlates with gene expression level

**Fig. 5.**
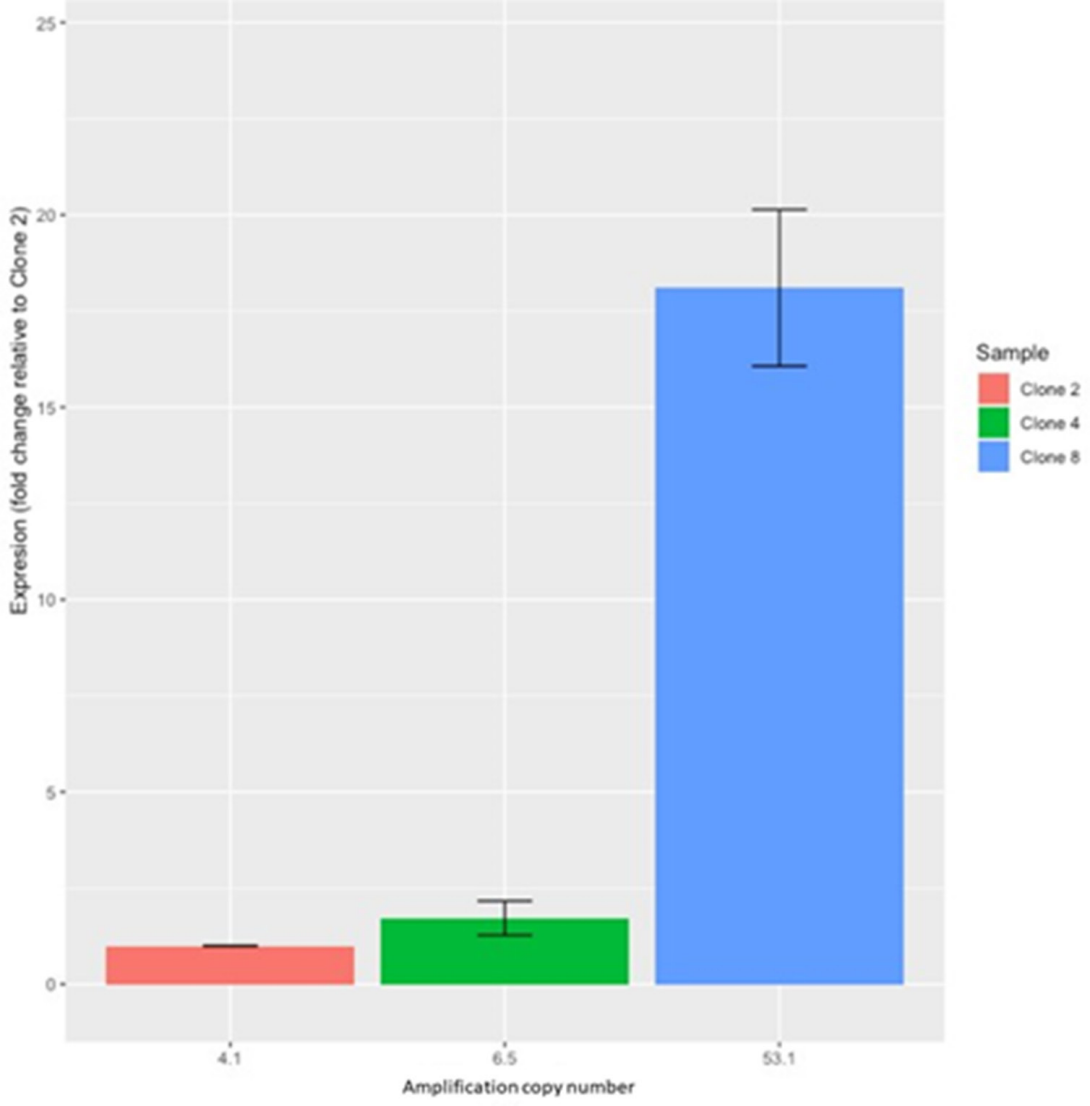
DNA copy number and RNA expression of the gene B1917_RS10525 was quantified in clones 2, 4 and 8 using qPCR and RT-qPCR, respectively. Expression is shown as a relative fold change to clone 2. Error bars represent standard deviation. The results show that copy number corresponds to gene expression levels.

To investigate the effect of copy-number variation on gene expression, we measured mRNA levels for one gene (B1917_RS10525 in B1917/BP1746 in Tohama) within the amplified locus in UK54 clones with different average copy numbers. Levels were normalized to the single-copy gene *recA* that is often used as a stably expressed housekeeping gene in RT-qPCR experiments. We selected clones 2, 4 and 8, with screened copy numbers of 2.63, 4.32 and 51.21, respectively. As we demonstrated that each culture comprises a heterogenous mixture of cells with varied amplification copy number, we re-estimated the locus copy number for each clone using the same laboratory culture from which RNA was extracted. Upon re-growing these clones for RNA extraction, the average copy number in each changed (non-significantly) to 4.1, 6.5 and 53.1 in clones 2, 4 and 8, respectively. The relative mRNA level for the amplified gene correlated with the copy number (Fig. 5); normalizing the transcript level in clone 2 to a value of 1, it was 16.8-fold higher (*P*<0.0001) in clone 8. It was also higher, but not significantly, in clone 4 (*P*=0.76). However, broadly, there was an association between gene copy number and transcript abundance. To investigate this further we chose three other genes from this locus, corresponding to different apparent transcriptional units across the locus as judged by gene organisation. We quantified the level of BP1736, BP1740 and BP1744 relative to *recA* in clones 3 and 8. We grew fresh cultures in triplicate of both clones for this and so verified the copy number of the locus in these cultures. The copy number of the locus in the three cultures of clone 3 was between 3 and 4, and for clone 10 all three were over 50. Relative to the level of expression in clone 3, expression of 1736, 1740 and 1744 in clone 10 was 13.76- (+/-1.69), 23.33- (+/-1.76) and 21.34- (+/-1.29) fold higher. This strongly suggests that the gene dosages produced by amplification of this locus affected relative gene expression levels within the cell.

### The presence of large amplifications in *Mycobacterium tuberculosis*

**Fig. 6.**
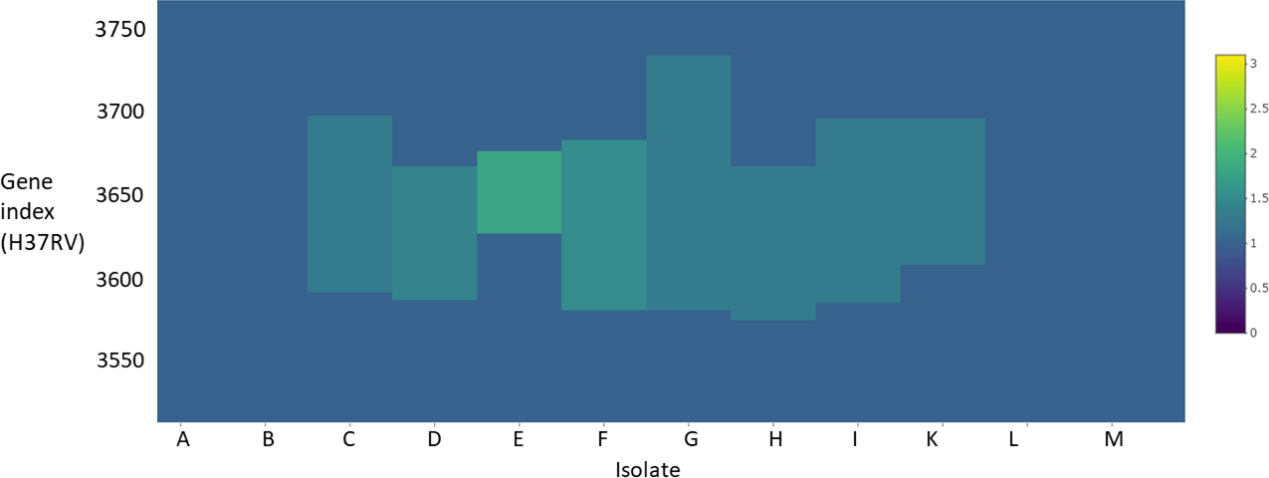
Heatmap of genes ~3550 to ~3750 (*Y* axis) in 12 isolates of *M. tuberuclosis* (*X* axis). Estimates of copy number are colour coded. Multiple regions of increased copy number can be seen, displaying a hotspot-like effect.

We sampled 1000 *M *. *tuberculosis* genome sequences reported by Walker *et al*. [46] and mapped to the reference genome sequence of H37Rv. Out of 1000 isolates, 945 had read-depth coverage that was consistent enough to predict amplifications. We identified 590 amplifications in 125 isolates. Similar to *B. pertussis *, 97 % of the amplifications were found at 24 hotspots (regions amplified in three or more isolates) (Fig. 6, Tables S4 and S5). It was apparent that 48 % (*n*=61) of these isolates had two or more amplifications (mean: 8.3) whereas this was 27 % (*n*=53) (mean: 5) in *B. pertussis *. Like *B. pertussis,* predicted copy numbers were largely non-integer suggesting they derived from sequencing a mixed population of cells.

The putative functions of duplicated genes were analysed. Genes were binned into likely ontological groups based on their homology to genes of known function using the COG ontology scheme (Table S6). There was no clear COG category that was over-represented among duplicated genes. However, we noted that genes encoding toxin-antitoxins (TA) were present in many of the duplications. Ramage *et al*. confirmed that at least 33 of the 80 putative TA systems that have been identified in *M. tuberculosis* were toxic in *M. smegmatis* [47]. Of these 33, 26 were found in the hotspot networks. Enrichment testing using Fisher's exact test found this enrichment was significant (*P*=0.048). Networks 1, 2, 3, 4, 7, 9, 15, 17 and 23 contained between one and eight active TA systems. However, when analysing for enrichment of TA systems in hotspot cores, which we suggest are the focus of selection for duplications (calculated only for networks with over ten isolates), there was no significant enrichment (*P*=0.2).

In network 1, the locus most frequently amplified, the *espACD* operon is at the centre of the hotspot and is amplified in 29 isolates. This gene cluster codes for proteins, which are essential for the ESX-1 (ESAT-6 system 1) type 7 protein secretion system that exports pro-inflammatory proteins involved in cell entry, phagosome escape and intercellular spread. Mutants of this locus demonstrated attenuated virulence in mice [48] but it is unclear how overexpression, the presumed transcriptional effect of amplification, might affect virulence.

Network 3 contained the *recBCD* operon, which codes for the RecBCD holoenzyme involved in double strand break DNA repair. In contrast to other species, however, RecBCD is not involved in homologous recombination in the species [49]. Overexpression of these genes is likely to alter in the ability of the cells to repair double-stranded breaks in some way.

## Discussion

We systematically analysed amplifications in both *B. pertussis* and *M. tuberculosis *, revealing there was greater genetic diversity in both species than previously thought. This contributes to a growing literature demonstrating that quantifying diversity requires a comprehensive view of mutation types, not just the quantification of DNA base changes and gene gain/loss [7, 31, 43, 50].

The highly repetitive *B. pertussis* genome suggests that a very large number of different genome rearrangements [7, 51] and amplifications are possible – yet they were primarily observed at just 11 loci. This disparity was striking. The observed frequency of an amplification is a product of its rate of formation, rate of loss and the difference of selective forces acting on the natural and amplified state [44, 52]. Given that the frequency of repeat regions in a genomic locus is the best described factor of local rates of amplification formation and loss [53] and that *B. pertussis* has relatively evenly spaced repeats [5], we hypothesize that selection was a major driver of the observed pattern of amplifications. We suggest that amplifications in hotspots are likely to be at an appreciable frequency as they are under neutral or positive selection. For the amplifications identified in *B. pertussis,* we described a number of functional categories of genes in the cores of hotspot loci, which may have been under positive selection, a pattern that was found in *Salmonella enterica* by Hjort *et al*. [2]. This included the genes of the flagella operons in hotspot 1, the most frequently observed amplification.

Motility has been frequently implicated in the virulence of bacterial pathogens, but *B. pertussis* has long been regarded as non-motile. However, recent research has shown that motility can be occasionally observed *in vitro* and flagellar biosynthesis genes are expressed during murine challenge compared to *in vitro* growth, potentially implicating motility or biofilm formation in infection [54]. Motility has been observed in only a small number of isolates and can occur sporadically [55]. The isolates found by Hoffman *et al*. to be motile do not have publicly accessible genome sequences and therefore it could not be established if these isolates contained amplifications at the flagella locus. While it is unclear as to the factors that determine when *B. pertussis* is motile, it is tempting to speculate that amplification of the flagella locus affects *B. pertussis* motility.

Multiple metabolic functions were affected by amplifications in our analysis including uptake of cystine and the electron transport chain. *B. pertussis* is auxotrophic for cysteine, potentially as several genes involved in sulphate transport and cysteine biosynthesis are present as pseudogenes and require trace amounts in laboratory medium for growth. It is possible that amplification of the genes for this predicted cystine import system increase the level of cystine uptake, providing a growth advantage. Interestingly, the genes encoding NADH:quinone oxidoreductase (*nuo*) have been implicated previously in an amplification hotspot [2], although it was found that in the presence of antibiotics, the *nuo* operon was not under positive selection, but hitchhiking. It is plausible that increased NADH:quinone oxidoreductase in *B. pertussis* aids the generation of ATP and therefore impacts fitness.

The instability of amplifications was exemplified by the observation of a UK54 clone with an average copy number of 51. In experimental systems, copy numbers of genes of up to 100 have been generated [56] and in clinically derived isolates the copy numbers of antimicrobial resistance genes can change rapidly, increasing up to 70 copies in response to antibiotics [1]. Whilst the function of the genes in the UK54 amplifications are unclear it is possible that they provide a fitness benefit to the isolate under certain conditions.

Our investigation demonstrates widely applicable approaches to the study of amplifications. Our application of a CNVnator-based pipeline utilizes short-read sequencing data that is available for thousands of bacteria. A limitation of this approach is that reads were mapped to B1917 and therefore amplifications were predicted as if the gene order was the same in B1917, despite frequent rearrangements in the population. This may lead to the read-depth signal being ‘split’ on the B1917 reference when they are, in truth, contiguous on another genome, leading to multiple amplification predictions. To overcome the limitations of short-read sequencing we therefore used long-read DNA sequencing for spanning repeat regions to enable resolution of amplifications and genome arrangement, although correct assemblies required additional data. However, genotypes could be described using single Nanopore reads to identify copy number heterogeneity within *in vitro* cultures.

Whilst unusual, *B. pertussis* is not unique in the abundance of its IS elements as it ranks in the top 30 in a study of 1000s of bacterial isolates [57]. Indeed, genomes of related species *B. parapertussis* and *B. holmesii* each harbour fewer IS elements and thus exhibit fewer rearrangements [51] and very rare amplifications (unpublished). This suggests that the vast repertoire of short-read sequence data for the bacterial kingdom is suited to high-throughput screening for amplifications, as our investigations in *M. tuberculosis* found [25, 58].

Previously, seven amplifications had been found at three loci in *M. tuberculosis* [24–26]. Only one of these shared a 50 % overlap with the amplifications identified here. Therefore, our data greatly expands the range of known amplifications for *M. tuberculosis *, and supports our assertion that amplifications are widespread among bacteria and identifiable from short-read data archives. Genes that were in the core of the three most frequent hotspots in *M. tuberculosis* included *PPE56* in hotspot 1, *rseA* in hotspot 2 and the *recBCD* operon in hotspot 3, which have been implicated in intracellular growth [59], virulence regulation [60, 61] and DNA maintenance [49, 62], respectively. How amplification of these genes effects the pathogenicity of the species is unclear however, although overexpression (the likely effect of amplification) of these genes can shed light on the likely impacts. Overexpression of the antisigma factor RseA was associated with decreased expression of both the SigE sigma factor and its regulon, knockouts of which are associated with increased bacterial persistence and attenuation in the mouse model [63]. Thus, it is tempting to speculate that amplification of *rseA* affects latency in *M. tuberculosis *.

In conclusion, we have identified widespread amplifications in *B. pertussis* and *M. tuberculosis *, revealing a novel layer of diversity that should be considered when quantifying variation within these species. As structural variants are found ubiquitously in all organisms, these results impact many fields of study. These include epidemiology, where amplifications would disrupt PFGE typing, masking ancestral relationships, and the development of antibiotics, considering that amplifications of resistance genes are frequently described yet are still likely underestimates [1]. Our results scratch the surface of the diversity of these mutations in the bacterial kingdom, with many more species with repetitive genomes likely harbouring similar repertoires. In particular, our contemporary genomic study of circulating *B. pertussis* should signal the end to its designation as a monomorphic species [64].

## Supplementary Data

Supplementary material 1

Supplementary material 2

Supplementary material 3

Supplementary material 4

Supplementary material 5

Supplementary material 6

Supplementary material 7

Supplementary material 8

## References

[1] Nicoloff H, Hjort K, Levin BR, Andersson DI (2019). The high prevalence of antibiotic heteroresistance in pathogenic bacteria is mainly caused by gene amplification. Nat Microbiol.

[2] Hjort K, Nicoloff H, Andersson DI (2016). Unstable tandem gene amplification generates heteroresistance (variation in resistance within a population) to colistin in *Salmonella enterica*. Mol Microbiol.

[3] Cerquetti M (2005). Multiple capsule genes in *H. influenzae*. JID.

[4] Mekalanos JJ (1983). Duplication and amplification of toxin genes in *Vibrio cholerae*. Cell.

[5] Parkhill J, Sebaihia M, Preston A, Murphy LD, Thomson N (2003). Comparative analysis of the genome sequences of *Bordetella pertussis*, *Bordetella parapertussis* and *Bordetella bronchiseptica*. Nat Genet.

[6] Xu Z, Wang Z, Luan Y, Li Y, Liu X (2019). Genomic epidemiology of erythromycin-resistant *Bordetella pertussis* in China. Emerg Microbes Infect.

[7] Weigand MR, Peng Y, Loparev V, Batra D, Bowden KE (2017). The History of *Bordetella pertussis* genome evolution includes structural rearrangement. J Bacteriol.

[8] King AJ, van Gorkom T, van der Heide HGJ, Advani A, van der Lee S (2010). Changes in the genomic content of circulating *Bordetella pertussis* strains isolated from the Netherlands, Sweden, Japan and Australia: adaptive evolution or drift?. BMC Genomics.

[9] Lam C, Octavia S, Sintchenko V, Gilbert GL, Lan R (2014). Investigating genome reduction of *Bordetella pertussis* using a multiplex PCR-based reverse line blot assay (mPCR/RLB). BMC Res Notes.

[10] Weigand MR, Peng Y, Cassiday PK, Loparev VN, Johnson T (2017). Complete genome sequences of *Bordetella pertussis* isolates with novel pertactin-deficient deletions. Genome Announc.

[11] Amman F, D’Halluin A, Antoine R, Huot L, Bibova I (2018). Primary transcriptome analysis reveals importance of IS elements for the shaping of the transcriptional landscape of *Bordetella pertussis*. RNA Biology.

[12] Brinig MM, Cummings CA, Sanden GN, Stefanelli P, Lawrence A (2020). Significant gene order and expression differences in *Bordetella pertussis* despite limited gene content variation. J Bacteriol.

[13] Dienstbier A, Pouchnik D, Wildung M, Amman F, Hofacker IL (2018). Comparative genomics of Czech vaccine strains of *Bordetella pertussis*. Pathog Dis.

[14] Caro V, Hot D, Guigon G, Hubans C, Arrivé M (2006). Temporal analysis of French *Bordetella pertussis* isolates by comparative whole-genome hybridization. Microbes Infect.

[15] Dalet K, Weber C, Guillemot L, Njamkepo E, Guiso N (2004). Characterization of adenylate cyclase-hemolysin gene duplication in a *Bordetella pertussis* isolate. Infect Immun.

[16] Heikkinen E, Kallonen T, Saarinen L, Sara R, King AJ (2007). Comparative genomics of *Bordetella pertussis* reveals progressive gene loss in Finnish strains. PLoS One.

[17] Weigand MR, Peng Y, Loparev V, Johnson T, Juieng P (2016). Complete genome sequences of four *Bordetella pertussis* vaccine reference strains from serum institute of India. Genome Announc.

[18] Weigand MR, Pawloski LC, Peng Y, Ju H, Burroughs M (2018). Screening and genomic characterization of filamentous hemagglutinin-deficient *Bordetella pertussis*. Infect Immun.

[19] Ring N, Abrahams JS, Jain M, Olsen H, Preston A (2018). Resolving the complex *Bordetella pertussis* genome using barcoded nanopore sequencing. Microb Genom.

[20] Smith I (2003). *Mycobacterium tuberculosis* pathogenesis and molecular determinants of virulence. Clin Microbiol Rev.

[21] Cohen KA, Manson AL, Desjardins CA, Abeel T, Earl AM (2019). Deciphering drug resistance in *Mycobacterium tuberculosis* using whole-genome sequencing: progress, promise, and challenges. Genome Med.

[22] Brennan MJ (2017). The Enigmatic PE/PPE multigene family of mycobacteria and tuberculosis vaccination. Infect Immun.

[23] Cole ST, Brosch R, Parkhill J, Garnier T, Churcher C (1998). Deciphering the biology of *Mycobacterium tuberculosis* from the complete genome sequence. Nature.

[24] Domenech P, Rog A, Moolji J, Radomski N, Fallow A (2014). Origins of a 350-kilobase genomic duplication in *Mycobacterium tuberculosis* and its impact on virulence. Infect Immun.

[25] Weiner B, Gomez J, Victor TC, Warren RM, Sloutsky A (2012). Independent large scale duplications in multiple *M. tuberculosis* lineages overlapping the same genomic region. PLoS One.

[26] Brosch R, Gordon SV, Garnier T, Eiglmeier K, Frigui W (2007). Genome plasticity of BCG and impact on vaccine efficacy. Proc Natl Acad Sci U S A.

[27] Zeddeman A, van Gent M, Heuvelman CJ, van der Heide HG, Bart MJ (2014). Investigations into the emergence of pertactin-deficient *Bordetella pertussis* isolates in six European countries, 1996 to 2012. Eurosurveillance.

[28] Li H (2013). Aligning sequence reads, clone sequences and assembly contigs with bwa-membwa-mem. arXiv.

[29] Abyzov A, Urban AE, Snyder M, Gerstein M (2011). CNVnator: an approach to discover, genotype, and characterize typical and atypical CNVs from family and population genome sequencing. Genome Res.

[30] Huang W, Li L, Myers JR, Marth GT (2012). ART: a next-generation sequencing read simulator. Bioinformatics.

[31] Bowden KE, Weigand MR, Peng Y, Cassiday PK, Sammons S (2016). Genome structural diversity among 31 *Bordetella pertussis* isolates from two recent U.S. whooping cough statewide epidemics. mSphere.

[32] Weigand MR, Pawloski LC, Peng Y, Ju H, Burroughs M (2018). Screening and genomic characterization of filamentous hemagglutinin-deficient *Bordetella pertussis*. Infect Immun.

[33] Csardi G, Nepusz T (2006). The igraph software package for complex network research. InterJournal Complex Systems.

[34] Fruchterman TMJ, Reingold EM (1991). Graph drawing by force-directed placement. Softw: Pract Exper.

[35] Minh BQ, Schmidt HA, Chernomor O, Schrempf D, Woodhams MD (2020). IQ-TREE 2: New Models and Efficient Methods for Phylogenetic Inference in the Genomic Era. Mol Biol Evol.

[36] Hoang DT, Chernomor O, von Haeseler A, Minh BQ, Vinh LS (2018). UFBoot2: Improving the Ultrafast Bootstrap Approximation. Mol Biol Evol.

[37] Minh BQ, Nguyen MAT, von Haeseler A (2013). Ultrafast approximation for phylogenetic bootstrap. Mol Biol Evol.

[38] Kalyaanamoorthy S, Minh BQ, Wong TKF, von Haeseler A, Jermiin LS (2017). ModelFinder: fast model selection for accurate phylogenetic estimates. Nat Methods.

[39] Letunic I, Bork P (2007). Interactive Tree Of Life (iTOL): an online tool for phylogenetic tree display and annotation. Bioinformatics.

[40] Ekblom R, Smeds L, Ellegren H (2014). Patterns of sequencing coverage bias revealed by ultra-deep sequencing of vertebrate mitochondria. BMC Genomics.

[41] Loman NJ, Misra RV, Dallman TJ, Constantinidou C, Gharbia SE (2012). Performance comparison of benchtop high-throughput sequencing platforms. Nat Biotechnol.

[42] Zhang L, Bai W, Yuan N, Du Z (2019). Comprehensively benchmarking applications for detecting copy number variation. PLoS Comput Biol.

[43] Weigand MR, Pawloski LC, Peng Y, Ju H, Burroughs M (2018). Screening and genomic characterization of filamentous hemagglutinin-deficient *Bordetella pertussis*. Infect Immun.

[44] Andersson DI, Hughes D (2009). Gene amplification and adaptive evolution in bacteria. Annu Rev Genet.

[45] Weidner U, Geier S, Ptock A, Friedrich T, Leif H (1993). The gene locus of the proton-translocating NADH: ubiquinone oxidoreductase in *Escherichia coli*. Organization of the 14 genes and relationship between the derived proteins and subunits of mitochondrial complex I. J Mol Biol.

[46] Walker TM, Kohl TA, Omar SV, Hedge J, Del Ojo Elias C (2015). Whole-genome sequencing for prediction of *Mycobacterium tuberculosis* drug susceptibility and resistance: a retrospective cohort study. Lancet Infect Dis.

[47] Ramage HR, Connolly LE, Cox JS (2009). Comprehensive functional analysis of *Mycobacterium tuberculosis* toxin-antitoxin systems: implications for pathogenesis, stress responses, and evolution. PLoS Genet.

[48] Fortune SM, Jaeger A, Sarracino DA, Chase MR, Sassetti CM (2005). Mutually dependent secretion of proteins required for mycobacterial virulence. Proc Natl Acad Sci U S A.

[49] Gupta R, Barkan D, Redelman-Sidi G, Shuman S, Glickman MS (2011). Mycobacteria exploit three genetically distinct DNA double-strand break repair pathways. Mol Microbiol.

[50] Weigand MR, Peng Y, Loparev V, Batra D, Bowden KE (2016). Complete genome sequences of four different *Bordetella* sp. isolates causing human respiratory infections. Genome Announc.

[51] Weigand MR, Peng Y, Batra D, Burroughs M, Davis JK (2019). Conserved patterns of symmetric inversion in the genome evolution of bordetella respiratory pathogens. mSystems.

[52] Pettersson ME, Sun S, Andersson DI, Berg OG (2009). Evolution of new gene functions: simulation and analysis of the amplification model. Genetica.

[53] Anderson P, Roth J (1981). Spontaneous tandem genetic duplications in *Salmonella typhimurium* arise by unequal recombination between rRNA (rrn) cistrons. Proc Natl Acad Sci U S A.

[54] van Beek LF, de Gouw D, Eleveld MJ, Bootsma HJ, de Jonge MI (2018). Adaptation of *Bordetella pertussis* to the respiratory tract. J Infect Dis.

[55] Hoffman CL, Gonyar LA, Zacca F, Sisti F, Fernandez J (2019). *Bordetella pertussis* can be motile and express flagellum-like structures. mBio.

[56] Edlund T, Grundström T, Normark S (1979). Isolation and characterization of DNA repetitions carrying the chromosomal beta-lactamase gene of *Escherichia coli* K-12. Mol Gen Genet.

[57] Robinson DG, Lee M-C, Marx CJ (2012). OASIS: an automated program for global investigation of bacterial and archaeal insertion sequences. Nucleic Acids Res.

[58] Yang F, Yang J, Zhang X, Chen L, Jiang Y (2005). Genome dynamics and diversity of *Shigella* species, the etiologic agents of bacillary dysentery. Nucleic Acids Res.

[59] Mestre O, Hurtado-Ortiz R, Dos Vultos T, Namouchi A, Cimino M (2013). High throughput phenotypic selection of *Mycobacterium tuberculosis* mutants with impaired resistance to reactive oxygen species identifies genes important for intracellular growth. PLoS One.

[60] Wu QL, Kong D, Lam K, Husson RN (1997). A mycobacterial extracytoplasmic function sigma factor involved in survival following stress. J Bacteriol.

[61] Jensen-Cain DM, Quinn FD (2001). Differential expression of sigE by *Mycobacterium tuberculosis* during intracellular growth. Microb Pathog.

[62] Singh A (2017). Guardians of the mycobacterial genome: A review on DNA repair systems in *Mycobacterium tuberculosis*. Microbiology.

[63] Ando M, Yoshimatsu T, Ko C, Converse PJ, Bishai WR (2003). Deletion of *Mycobacterium tuberculosis* sigma factor E results in delayed time to death with bacterial persistence in the lungs of aerosol-infected mice. Infect Immun.

[64] Mooi FR (2010). *Bordetella pertussis* and vaccination: the persistence of a genetically monomorphic pathogen. Infect Genet Evol.

