# Supplementary material for: Towards comprehensive understanding of bacterial genetic diversity: large-scale amplifications in Bordetella pertussis and Mycobacterium tuberculosis
Source: Microb Genom. 2022 Feb 10;8(2):000761. doi: 10.1099/mgen.0.000761 (PMC8942028; doi:10.1099/mgen.0.000761)
Supplement: Supplementary material 1 [file mgen-8-0761-s001.pdf]

Supplementary figures

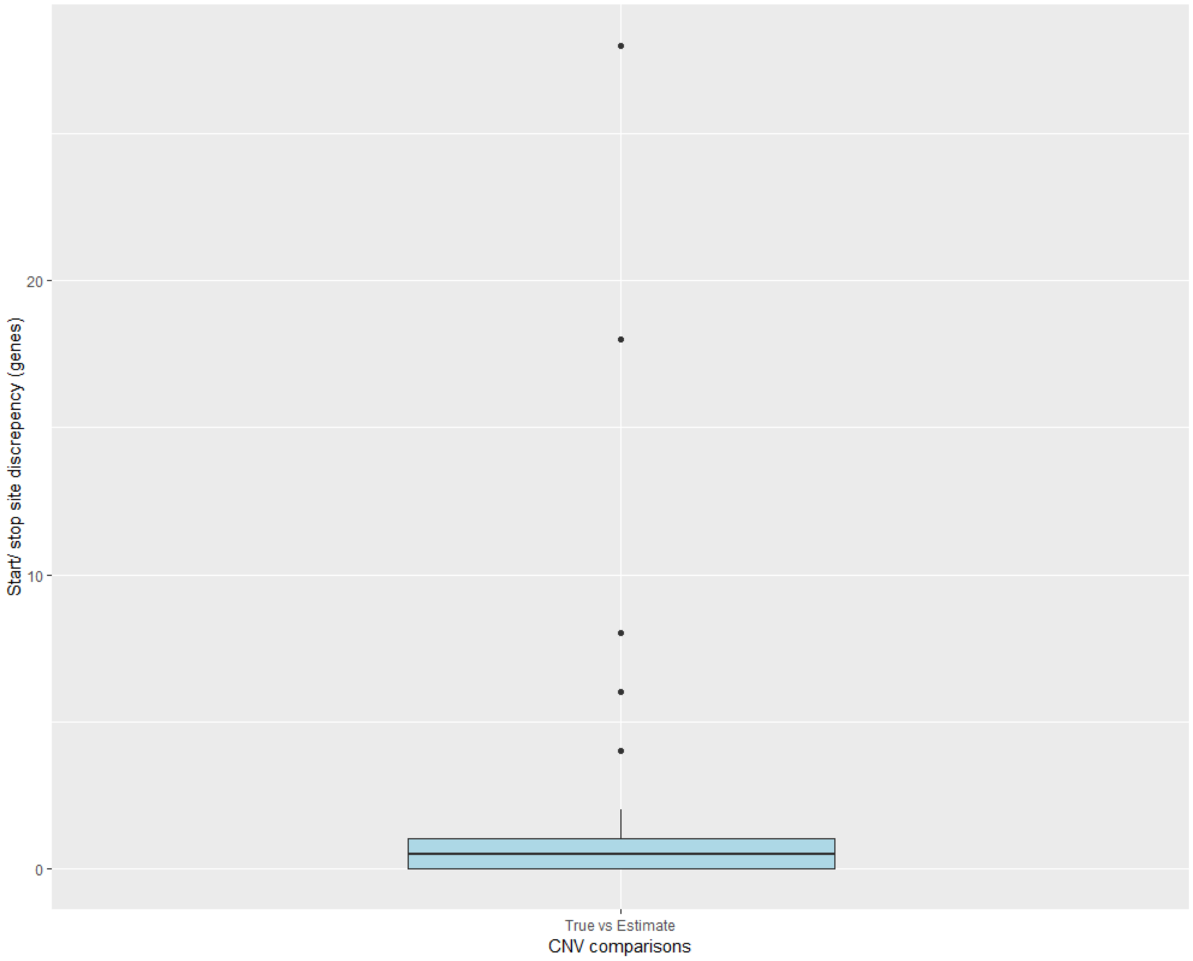

Supplementary Figure 1. The discrepancy between the start and end breakpoints of the 23 true positive predicted amplifications and the true amplifications was analysed as a boxplot. This showed a tight distribution around the median distance of 0.5 genes discrepancy.

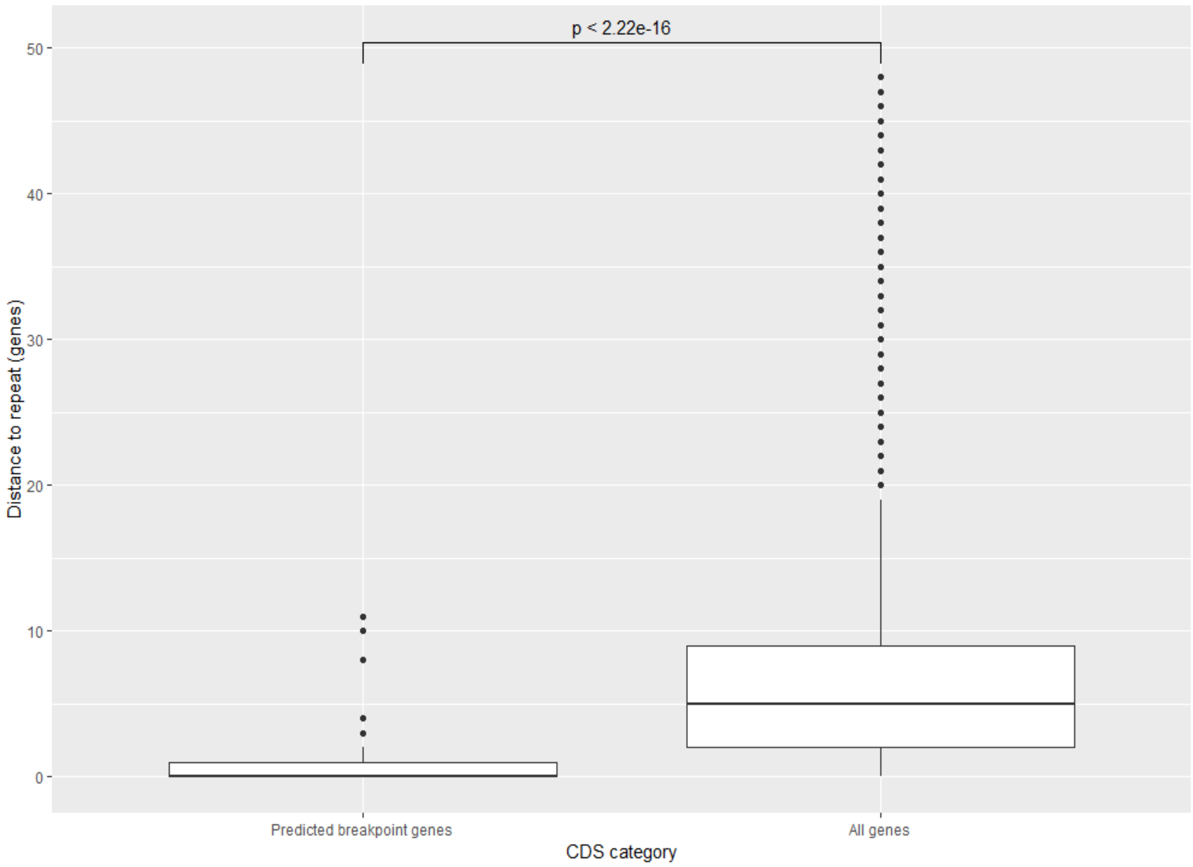

Supplementary Figure 2. The distance (measured in genes) between amplification start/end genes and repeat genes was identified in closed genomes. The ends of CNV loci were found to be significantly closer (median: 0 genes) to repeats than the average gene (median: 5 genes).

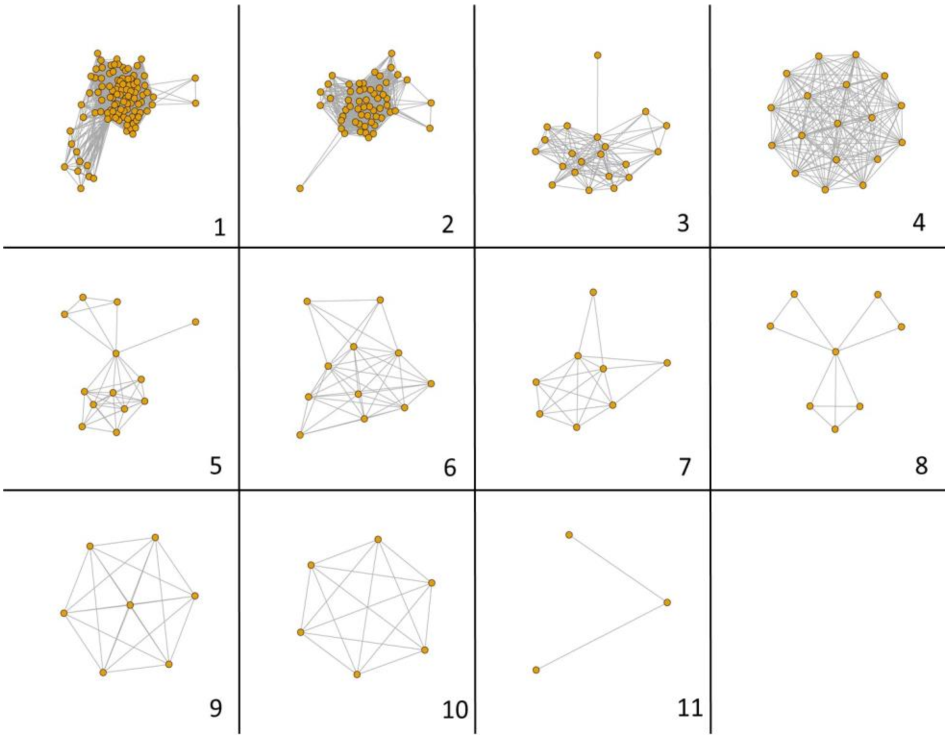

Supplementary Figure 3. Each network with 3 or more nodes (amplifications) was plotted as a graph which was arranged with the Fruchterman algorithm. Some probable communities can be seen within networks 1,5 and 8 whilst network 4 is clearly highly connected, as reflected in high network density.

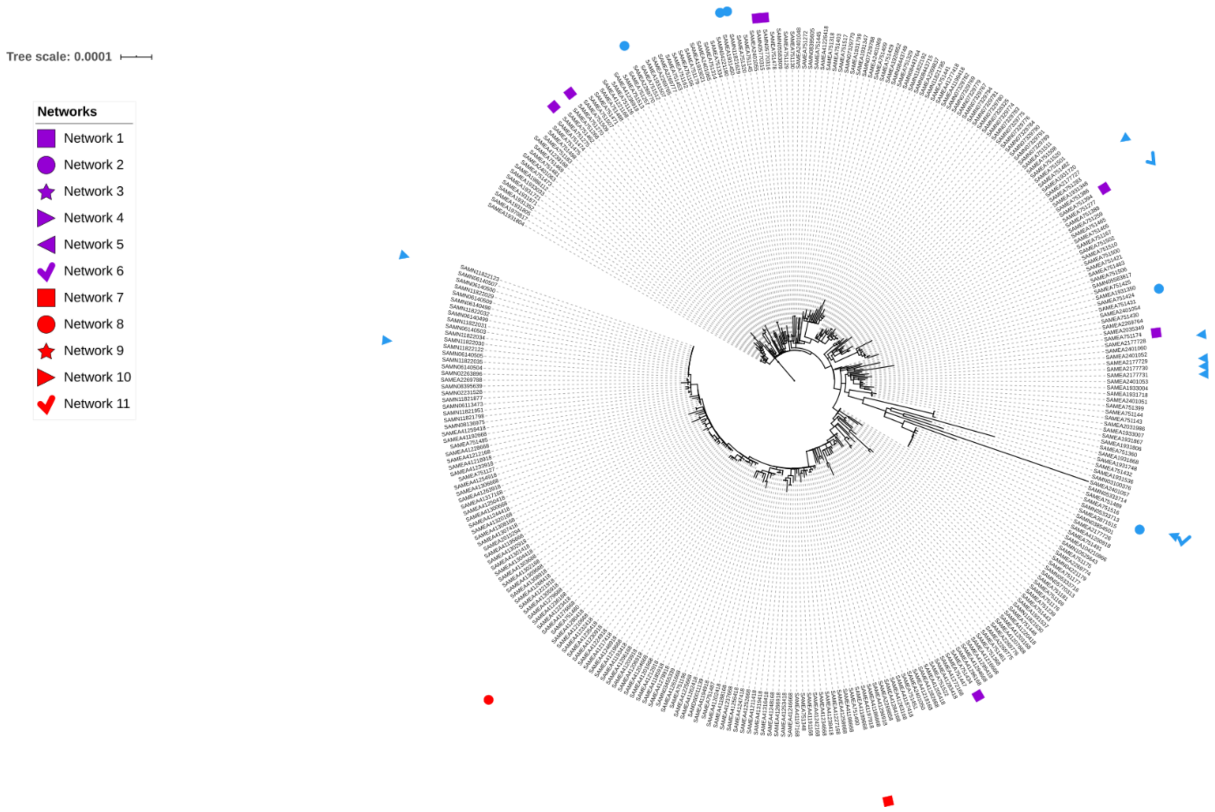

Supplementary Figure 4. A phylogenetic tree of all 2431 B. pertussis isolates was made. Shown here is a clade of just 317 isolates in order for branch lengths to be seen. Isolates containing amplifications belonging to the 11 networks of amplifications are annotated on the periphery of the tree. It can be seen that isolates containing amplifications from the same network were phylogenetically distant from one another. This indicates these mutations occurred independently, not from a single origin.

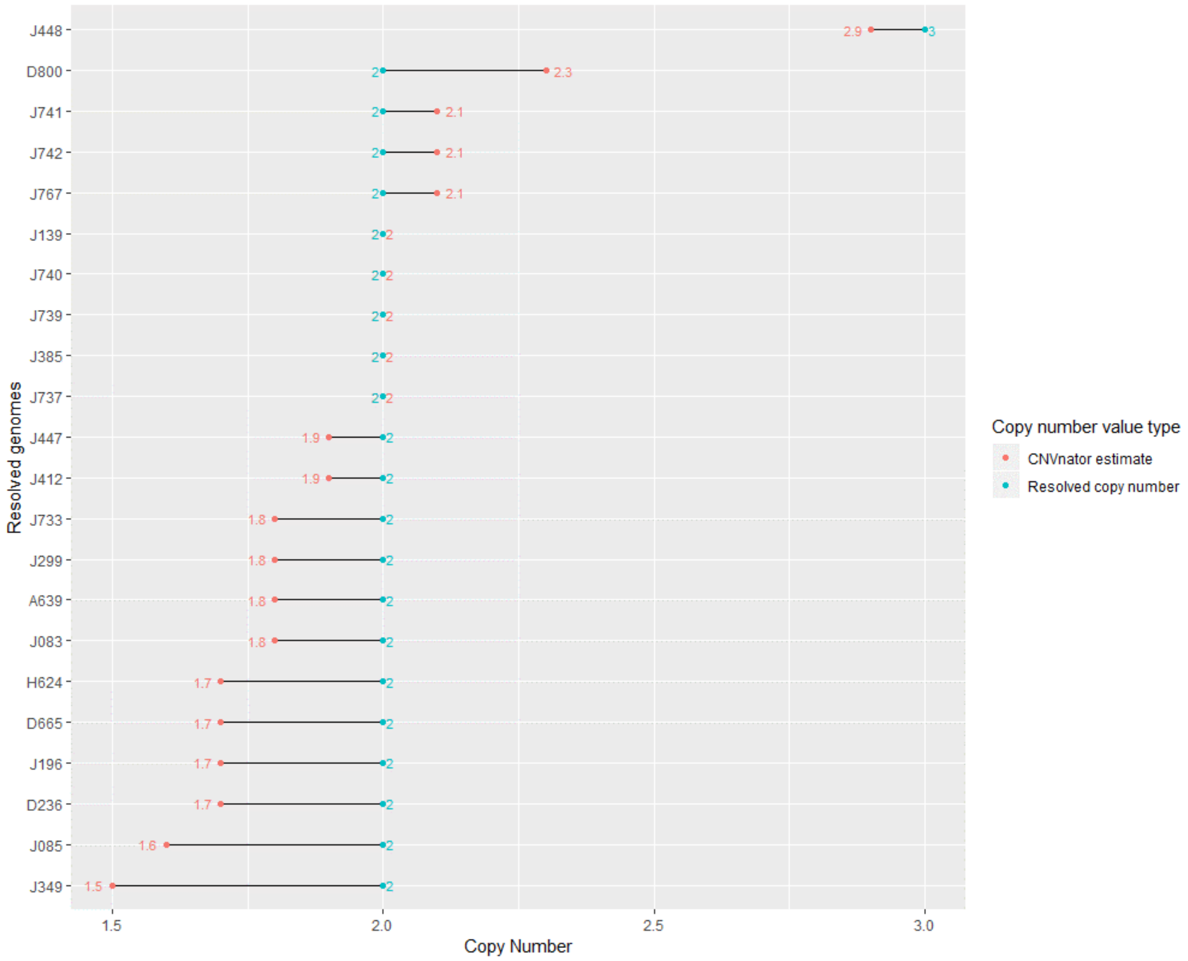

Supplementary Figure 5. Discrepancies between the copy number of amplifications predicted by CNVnator (orange values) and the resolved genome sequence copy number (blue values) in each isolate.
