# Supplementary material for: Towards comprehensive understanding of bacterial genetic diversity: large-scale amplifications in Bordetella pertussis and Mycobacterium tuberculosis
Source: Microb Genom. 2022 Feb 10;8(2):000761. doi: 10.1099/mgen.0.000761 (PMC8942028; doi:10.1099/mgen.0.000761)
Supplement: Supplementary material 8 [file mgen-8-0761-s008.pdf]

Supplementary Tables and Figure Legends.

Supplementary Table S1.

Manually resolved amplifications in 28 closed genomes were used as a dataset to test the specificity and sensitivity of the read depth-based method. Columns describe, from left to right: Alias of the strain (A), SRA ID of the run(B), estimated start of the amplification(C), estimated end of the amplification(D), estimated length of the amplification(E), estimated copy number (F), the resolved start(G), the difference between the predicted start and resolved start (H), the resolved end(I) , the difference between the predicted end and resolved end (J), the resolved copy number (K), a value determining if the estimated copy number was higher,lower or within 0.2 of the resolved copy number (L), if there was a reciprocal overlap (M) and if the prediction was a false positive (N). All gene indexes are in reference to B1917.

Supplementary Table S2.

A list of all amplifications in *B. pertussis*, their start/end genes, copy number, isolate and network.

Supplementary Table S3.

Expanded descriptions of all 24 networks of amplifications found in *B. pertussis* including frequency, mean length, median start/end/middle genes, mean copy number and isolates. Hotspot networks which have at least 10 constituent amplifications have their core (genes contained in >90% of amplifications in the network) described. The standard deviation of the start and end sites, measured in whole genes, is noted.

Supplementary Table S4.

A list of all amplifications in *M. tuberuclosis*, their start/end gene, copy number, isolate and network.

Supplementary Table S5.

Expanded descriptions of all 34 networks of amplifications found in *M. tuberculosis*, including frequency, mean length, median start/end/middle genes, mean copy number and isolates. Hotspot networks which have at least 10 constituent amplifications have their core (genes contained in >90% of amplifications in the network) described. The standard deviation of the start and end sites, measured in whole genes, is noted.

Supplementary Table S6.

Tables annotating reference genomes in *B. pertussis* and *M. tuberculosis* with functions and COG categories. For *B. pertussis*, conversion between B1917 genes and the more commonly used reference Tohama is included.

Supplementary Figure S1.

The discrepancy between the start and end breakpoints of the 23 true positive predicted amplifications and the true amplifications was analysed as a boxplot. This showed a tight distribution around the median distance of 0.5 genes discrepancy.

Supplementary Figure S2.

The distance (measured in genes) between amplification start/end genes and repeat genes was identified in closed genomes. The ends of CNV loci were found to be significantly closer (median: 0 genes) to repeats than the average gene (median: 5 genes).

Supplementary Figure 3.

Each network with 3 or more nodes (amplifications) was plotted as a graph which was arranged with the Fruchterman algorithm. Some probable communities can be seen within

networks 1,5 and 8 whilst network 4 is clearly highly connected, as reflected in high network density.

Supplementary Figure 4.

A phylogenetic tree of all 2431 *B. pertussis* isolates was made. Shown here is a clade of just 317 isolates in order for branch lengths to be seen. Isolates containing amplifications belonging to the 11 networks of amplifications are annotated on the periphery of the tree. It can be seen that isolates containing amplifications from the same network were phylogenetically distant from one another. This indicates these mutations occurred independently, not from a single origin.

Supplementary Figure 5.

Discrepancies between the copy number of amplifications predicted by CNVnator (orange values) and the resolved genome sequence copy number (blue values) in each isolate.
